# Cocoa and Chocolate

**Published:** 1887-03

**Authors:** 


COCOA AND CHOCOLATE. Published by Walter Baker & Co., Dorchester, Mass.

This charming little volume is intended to give a full account of the history, uses, and method of preparing this valuable food. It is issued by the manufacturers of chocolate. The account given of this interesting vegetable is quite full. It is beautifully printed in handsome type on fine paper. It is, of course, particularly interesting to physicians. W. M. J.

